# Transformation From Oxyntic Gland Neoplasm Into Advanced Gastric Cancer via *TP53* Mutation: A First Case Report

**DOI:** 10.14740/jmc5339

**Published:** 2026-09-04

**Authors:** Akiko Komatsu, Hirofumi Rokutan, Naoko Arai, Takuya Nagasaka, Seiya Kamino, Kazushi Fukagawa, Satoshi Ono, Nobuo Kanazawa, Tomio Arai

**Affiliations:** a Department of Pathology, Tokyo Metropolitan Institute for Geriatrics and Gerontology, Tokyo, Japan; b Department of Gastrointestinal and General Surgery, Tokyo Metropolitan Institute for Geriatrics and Gerontology, Tokyo, Japan; c Department of Gastroenterology and Gastrointestinal Endoscopy, Tokyo Metropolitan Institute for Geriatrics and Gerontology, Tokyo, Japan

**Keywords:** Gastric adenocarcinoma of fundic gland type, Oxyntic gland neoplasm, Transformation, *TP53*, Double stain

## Abstract

The molecular genetic mechanisms behind oxyntic gland neoplasm (OGN) progression are not well established. We present the first case suggesting that a *TP53* mutation caused the transformation from OGN into advanced gastric cancer with nodal metastasis. An 80-year-old man presented with anemia and was found to have a gastric tumor measuring 35 × 30 mm. Histology revealed predominantly invasive tubular adenocarcinoma showing diffuse p53 positivity, with a minor chief cell-predominant OGN component. Targeted sequencing of the entire tumor revealed a pathogenic *TP53* c.832C>A (p.Pro278Thr) mutation; no mutations were found in *GNAS* or *KRAS*. A double stain (p53/MUC5AC) provided immunohistochemical evidence of a shift from wild-type to mutant-type p53 expression, particularly in the foveolar epithelium within the intramucosal OGN component, suggesting the acquisition of a *TP53* mutation during tumor progression. This case supports an unreported role for *TP53* in the transformation of OGNs into aggressive carcinomas.

## Introduction

Gastric adenocarcinoma of the fundic gland type (GA-FG) is a rare cancer that typically originates from the fundic gland without atrophy. In the World Health Organization’s (WHO) tumor classification system, lesions with these histological features that remain in the mucosa are classified as oxyntic gland adenoma (OGA), while those with submucosal invasion are considered GA-FG [1]. Recently GA-FG with foveolar epithelium differentiation has been subclassified as gastric adenocarcinoma of fundic-gland mucosa type (GA-FGM). Collectively, OGA, GA-FG, and GA-FGM are referred to as oxyntic gland neoplasms (OGNs). Somatic mutations in *GNAS* and *KRAS* have previously been reported in OGNs [2–6]. However, previous findings regarding the role of the *TP53* gene or immunohistochemical overexpression of p53 in OGNs are limited [5–11].

We describe a case of *TP53*-mutated advanced gastric cancer—with nodal metastasis—originating from an OGN showing a normal (wild-type) p53 staining pattern. This report suggests an unreported molecular mechanism involved in the transformation from OGN to aggressive cancer.

## Case Report

An 80-year-old man visited our hospital for examination of anemia and exertional dyspnea. Upper gastrointestinal endoscopy revealed a tumor in the upper posterior wall of the gastric body. The degree of atrophic change in the background mucosa was type O1 according to the Kimura-Takemoto classification [12]. A histopathological examination of the tumor biopsy showed moderately to well-differentiated tubular adenocarcinoma. A computed tomography scan revealed metastasis of gastric cancer to lymph nodes along the lesser curvature of the stomach, but no distant metastasis to other organs. The patient underwent a total gastrectomy with D1 lymphadenectomy.

**Figure 1:**
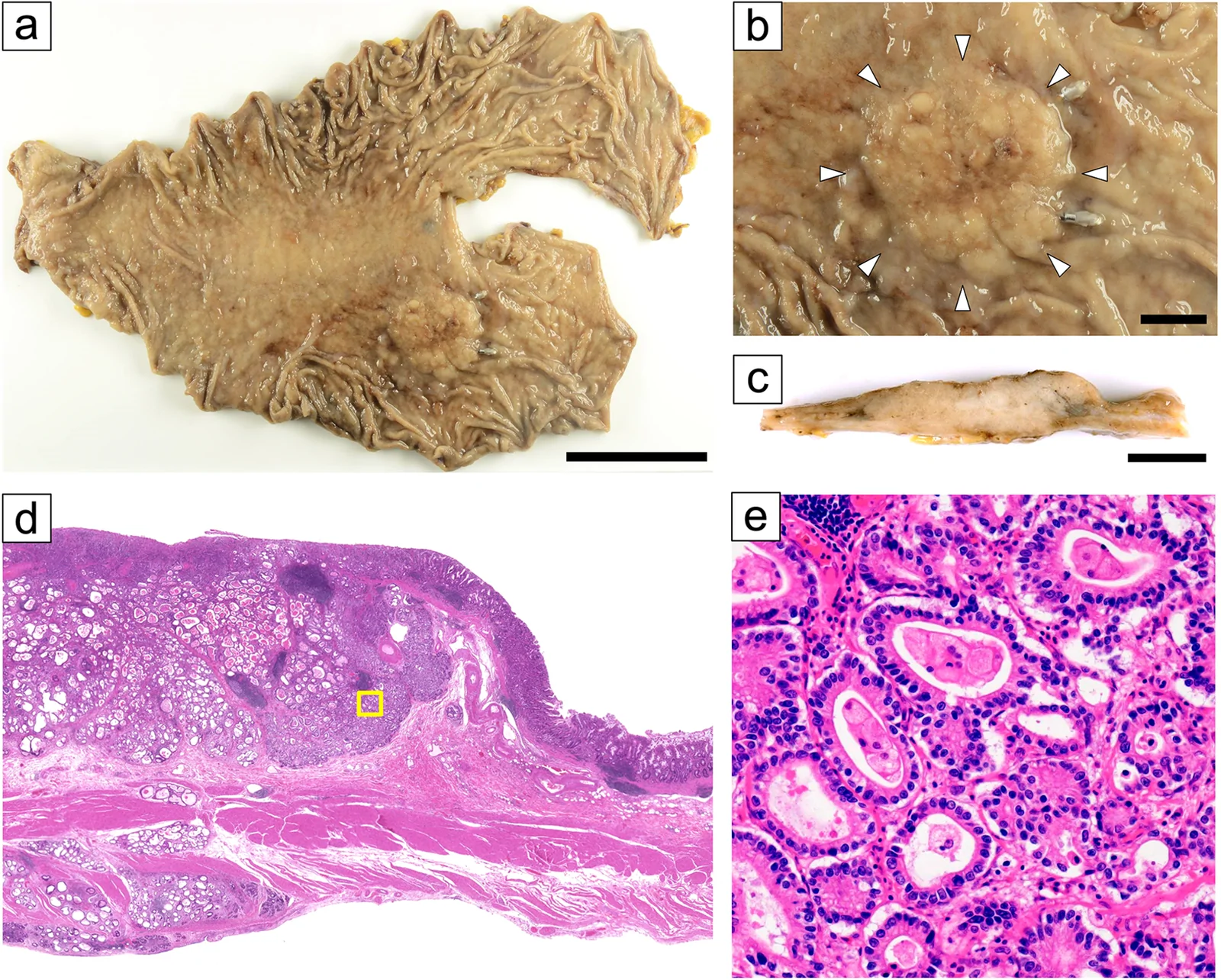
Macroscopic images of the resected specimen and histopathological findings of invasive component. (a) A tumor measuring 35 × 30 mm in size was observed on the posterior wall of the upper gastric body (scale bar: 5 cm). (b) The lesion borders were indistinct (arrow heads) (scale bar: 1 cm). (c) The cut surface of the tumor (scale bar: 1 cm). (d) Loupe view, tubular adenocarcinoma invading the subserosal layer. (e) A representative image of invasive tubular adenocarcinoma (yellow frame in d).

**Figure 2:**
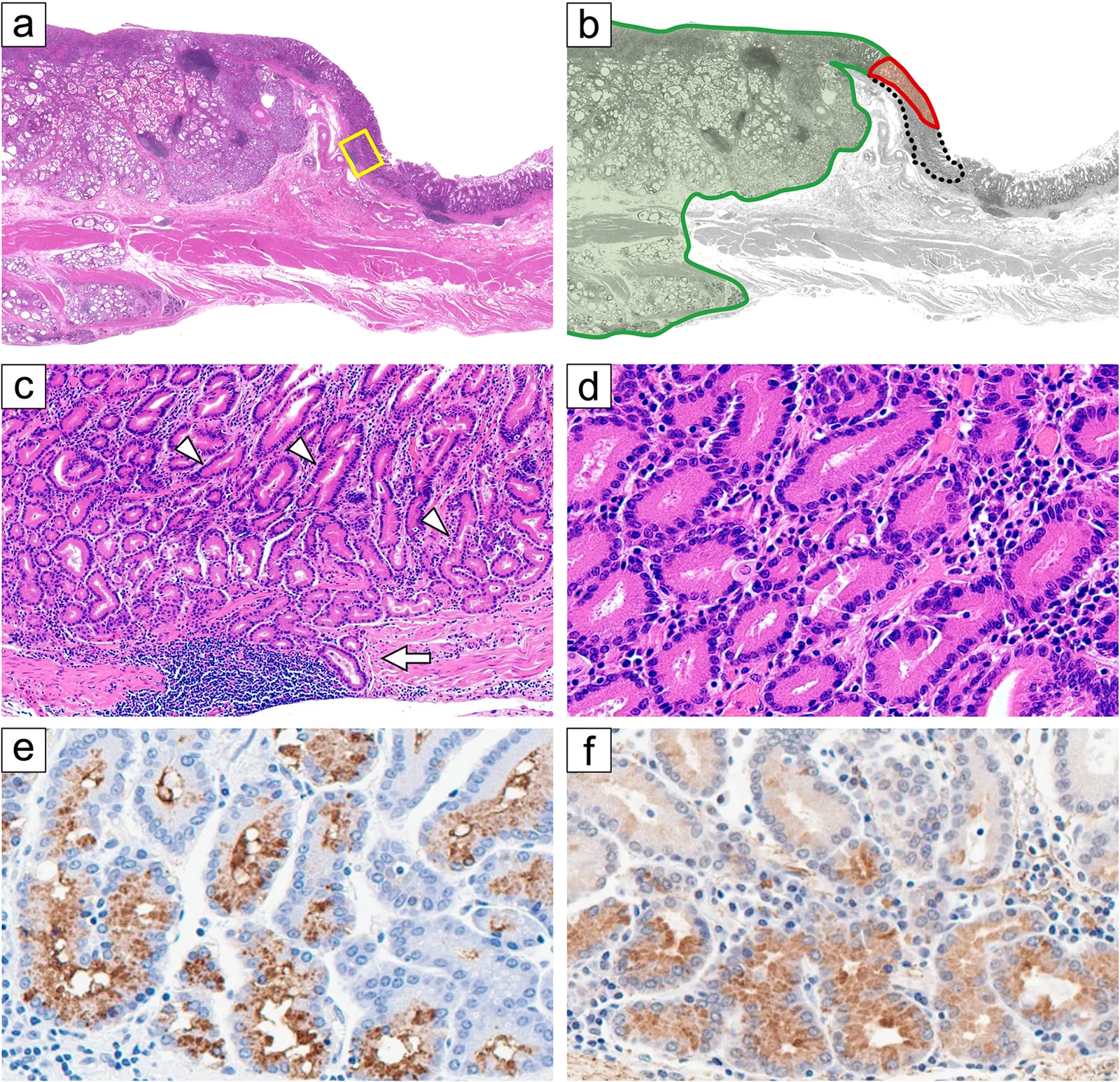
Histopathological findings with a focus on oxyntic gland neoplasm component. (H&E stain: a, c, d) (a, b) Loupe view. (b) This tumor consisted of three histological components: the largest proportion (> 90%) was intestinal-type adenocarcinoma (green area); we noted an oxyntic gland neoplasm (OGN) component with mild nuclear atypia in the edge of the mucosal component (surrounded by a black dashed line); the OGN component also contained an area (red) with nuclear atypia higher than in the dashed line area. (c) The OGN component with mild nuclear atypia (yellow frame in a; inside the black dashed area in b). Here we noted branching glands (arrowheads) and a small focus suggestive of pseudoinvasion (arrow) (d) The OGN component predominantly showed chief cell morphology, with positivity for MUC6 (e) and pepsin A (f).

**Figure 3:**
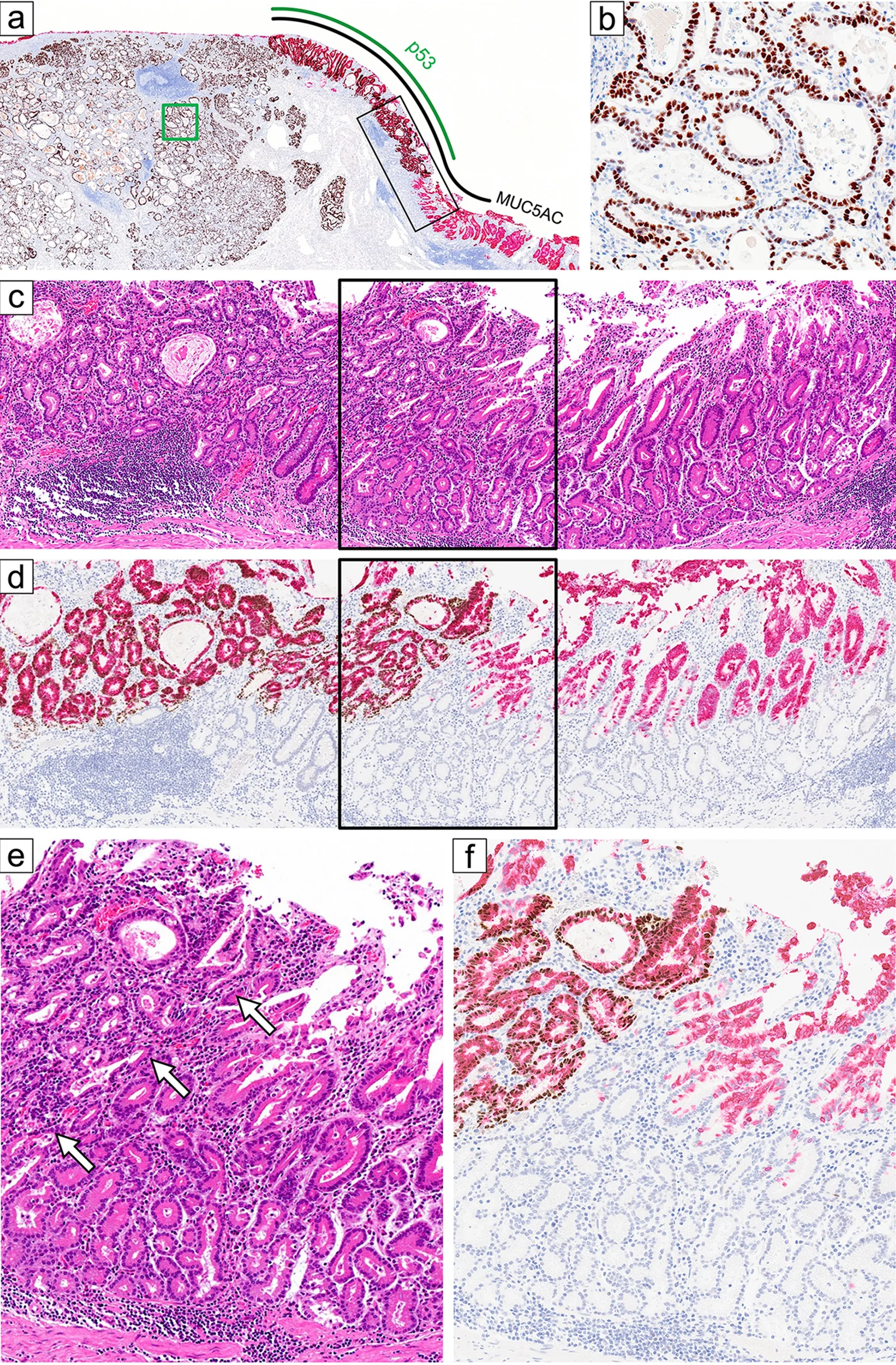
A detailed assessment of the role of *TP53* utilizing a p53/MUC5AC double stain. (a) Loupe view of double staining with MUC5AC (red) and p53 (brown). (b) High power view (red frame in a, double stain) of the massively invasive component (corresponding to Fig. 1e), showing diffuse p53 positivity. Low power panoramic view (c, d; black frame in a) and high power view (e, f) of the mucosal oxyntic gland neoplasm component. (d and f) Double stains with p53 (brown, nuclei) and MUC5AC (red, cytoplasmic). The surface of this area is covered by foveolar epithelium showing MUC5AC positivity (stained red). The area of e and f is indicated by black frames in c and d, respectively. A shift in the p53 staining pattern (brown in d and f) occurred within the foveolar component (stained red). This p53 immunohistochemical shift corresponds with the morphological shift (arrows). Note that the p53-positive area (left in f) shows a slightly higher grade of atypia (left in e) than in the other glands, with a 1.2–1.3-fold increase in cellularity and hyperchromasia. This border separates the red and black dashed line areas in Figure 2b.

Grossly, an elevated tumor measuring 35 × 30 mm in size was observed on the posterior wall of the upper gastric body (Fig. 1a–c). The gastric folds surrounding the tumor were slightly attenuated. The whole resected tumor was embedded in paraffin, and all the slides were evaluated. The dominant histology was moderately to well-differentiated tubular adenocarcinoma (Fig. 1d, e). The cancer had invaded the subserosal layer, and a poorly differentiated carcinoma was noted as a minor component in the submucosal and muscular layers. By thorough examination of the mucosal component, we noticed an OGN component (< 5% of the whole tumor; < 20% of the mucosal component) at the edge of the tumor (Fig. 2a–d). This OGN component primarily consisted of columnar cells with mild nuclear atypia, containing secretory vesicles and basally located nuclei (chief cell morphology). The component was intramucosal and had a very small focus suggestive of pseudoinvasion (Fig. 2c). The OGN component with chief cell morphology was covered with foveolar-type epithelial tissue on the surface (Fig. 3d; red staining represents MUC5AC). Thus, this OGN was subclassified as gastric adenocarcinoma of fundic-gland mucosa type (GA-FGM) [5, 9], in which foveolar epithelium differentiation is accompanied by fundic-gland differentiation.

The immunohistochemistry analyses are summarized in Table 1. Neoplastic columnar cells (with mild nuclear atypia) showing chief cell morphology in the OGN were positive for MUC6 and pepsin A, further supporting chief cell differentiation (Fig. 2e, f). H^+^/K^+^-ATPase-positive neoplastic cells were rare. MUC5AC was stained in the foveolar-type atypical neoplastic cells located on the surface of the tumor, confirming GA-FGM. Invasive adenocarcinoma component was also focally positive for MUC5AC. The intramucosal low-grade OGN component with chief cell predominance exhibited wild-type-pattern p53 staining. In contrast, high-grade atypical glands in the mucosa and massively invasive components showed diffuse positivity for p53 (Fig. 3a, b). A double immunohistochemical stain (p53/MUC5AC) highlighted an intramucosal p53(+)/MUC5AC(+) atypical foveolar epithelial component, just adjacent to the p53-wild-type neoplastic area with mild nuclear atypia (Fig. 3c–f); a shift in the p53 staining pattern occurred within the intramucosal foveolar component. Nuclear accumulation of β-catenin was not detected. The whole tumor was negative for HER2 and showed retained expression of MLH1.

**Table 1:** Immunohistochemical Results

	Oxyntic gland neoplasm component		Invasive component^a^
	Chief cell component	Foveolar-type epithelium	
MUC5AC	−	+	+ (focal)
MUC6^b^	+	−	−
Pepsin A^b^	+	−	−
p53	Wild-type pattern	Wild-type pattern → diffuse positive^c^	Diffuse positive
Nuclear β-catenin	−	−	−

^a^Virtually all of the invasive component showed tubular adenocarcinoma morphology. ^b^Positive only for the chief cell component of oxyntic gland neoplasm. ^c^The shift in p53 expression pattern is displayed in Figure 3c–f.

Vascular invasion and two lymph node metastases were also observed. The cancer cells in nodal metastasis exhibited diffuse p53 positivity and were negative for MUC5AC, MUC6, pepsin A, and H^+^/K^+^-ATPase. The tumor was pathologically staged as T3N1M0. Cancer relapse has not been observed after the resection (last follow-up: 25 months).

**Figure 4:**
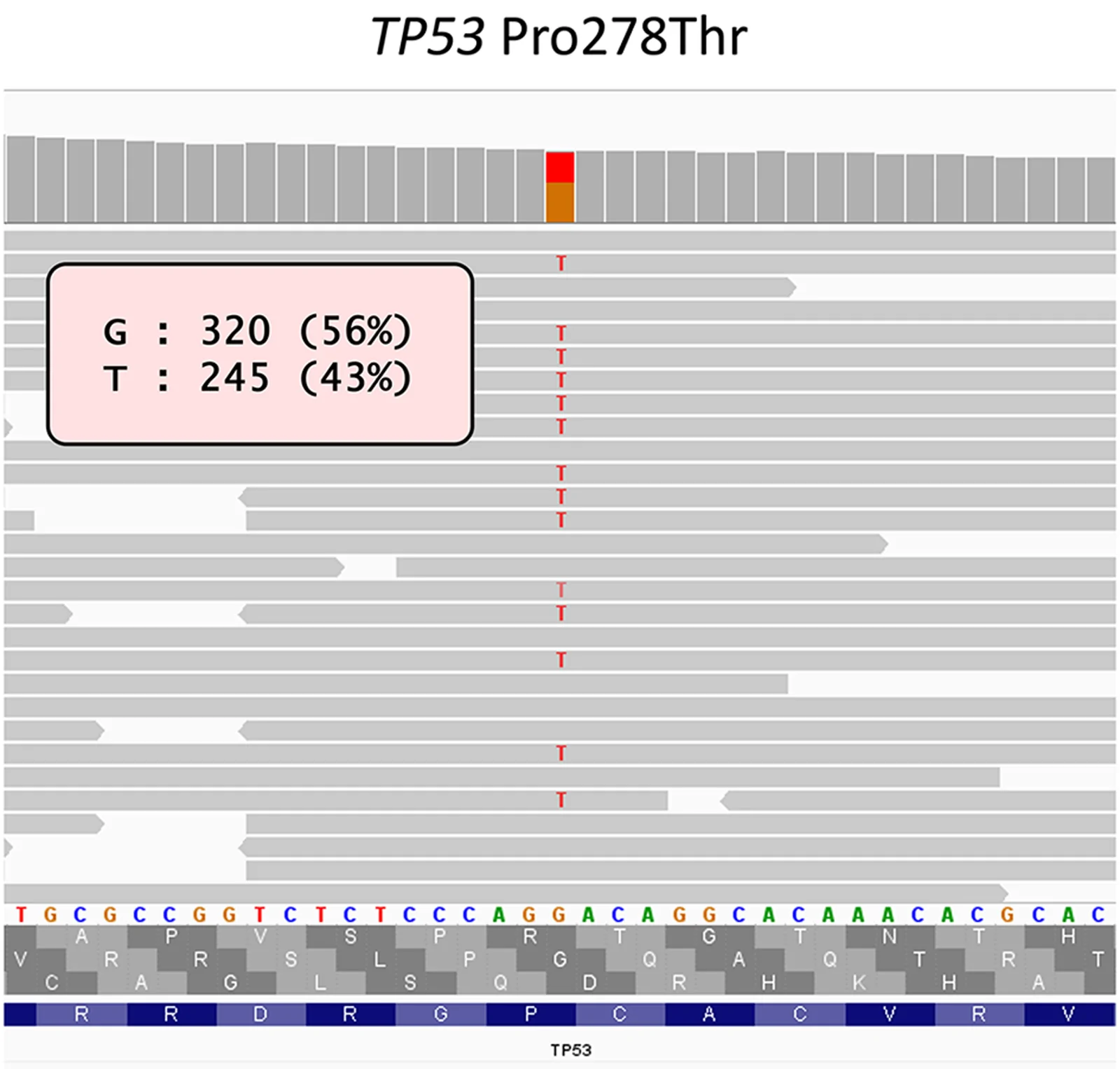
Image of the reads of the *TP53* mutation identified through sequencing 88 genes. Raw sequence data are displayed using the Integrative Genomics Viewer, showing *TP53* c.832C>A (p.Pro278Thr) mutation. The entire tumor lesion of the representative slide was subjected to next-generation DNA sequencing.

After obtaining the patient’s informed consent, the whole tumor area within a representative formalin-fixed paraffin-embedded slide was manually dissected (see Supplementary Material 1, jmc.elmerpub.com (tumor cellularity: 50%)), and genomic DNA was extracted. Targeted DNA sequencing was performed using Axen Cancer Panel 1 (Macrogen, Inc.), which covers 88 cancer-related genes (Supplementary Material 2, jmc.elmerpub.com). The Q30 ratio was 88.6% and the mean depth was 1,316. The filtering condition for variant detection was an allele frequency of > 10% and a depth of coverage of > 100×. Six exonic variants were identified in the tumor by next-generation sequencing analysis. Of them, only one variant showed pathogenicity: *TP53* c.832C>A (p. Pro278Thr) (Fig. 4), which is a mutation affecting the DNA-binding domain. The variant allele frequency at this *TP53* locus was 0.42, with a read depth of 314. No hotspot or pathogenic mutations were detected in other genes, including *GNAS*, *KRAS*, *APC*, and *CTNNB1*.

## Discussion

As relatively novel entities, the WHO first described OGA and GA-FG in the fifth edition of its tumor classification system [1]. Because of their slow growth and rare lymphovascular invasion, OGA and GA-FG (collectively called OGNs) generally have a favorable prognosis [5], but they occasionally show an aggressive clinical course. The first case of GA-FG with lymph node metastasis was reported in 2018 [8]. Advanced cases (pT2–T3) have also been recently reported [13], with an incidence of 14% (3/21). However, molecular events associated with the aggressive phenotype in OGNs have not been established to date. To our knowledge, this is the first case report showing molecular clue that transformation from GA-FGM into advanced gastric cancer was caused by *TP53* mutation, suggested by bulk DNA sequencing and p53/MUC5AC double immunohistochemistry.

Although p53 overexpression is predictive of a poor prognosis in gastric cancer patients [14], the role of p53 (or *TP53* gene) in OGNs has only been described to a limited extent [5–11]. In a large series by Ueyama et al [5], five of 70 GA-FGs exhibited focal p53 overexpression, but none harbored *TP53* mutations. A recent series by Liu et al [6] included one GA-FG with a *TP53* missense mutation among 10 sequenced GA-FGs. Liu et al did not provide details of the *TP53* mutation or intratumoral heterogeneity of p53 status. What we find interesting is that their *TP53*-mutated tumor showed the deepest infiltration among their 10 tumors. Although case numbers are few, our case and those of Liu et al suggest a critical role of *TP53* in GA-FGs in that *TP53*-mutated GA-FGs tend to invade deep into the gastric wall.

Another strength of this case report is the identification of a shift in p53 staining status—from a wild-type pattern to a mutant-type pattern—in the mucosal OGN component. We performed a p53/MUC5AC double immunohistochemical stain because determining when the *TP53* mutation was acquired in the tumor was of great interest. Our double stain suggested that the foveolar-type neoplastic component of GA-FGM had acquired a *TP53* mutation and the potential for aggressive transformation. Future studies investigating the correlation between GA-FGM and p53 (or *TP53* gene) are needed, because GA-FGMs are more commonly associated with submucosal and lymphovascular invasion compared with GA-FG [5].

According to prior studies, mutated genes in OGNs include *KRAS*, *GNAS*, and Wnt/β-catenin pathway genes. Ueyama et al [5] identified *GNAS* mutation in 21% (7/34) of OGNs, and suggested that OGA, GA-FG, and GA-FGM are spectral entities, as *GNAS* mutation was detected in all three entities. Less commonly, *KRAS* mutation is identified in 8.1% (5/62) of OGNs when calculated in total [3, 4, 6]. There are conflicting results regarding mutations in Wnt/β-catenin pathway genes [3, 6, 15], including *CTNNB1*, *APC*, *AXIN1*, and *AXIN2*, although accumulation of nuclear β-catenin is frequently reported [3]. Our tumor did not harbor mutations in *GNAS*, *KRAS*, and sequenced Wnt/β-catenin pathway genes (*CTNNB1*, *APC*). Nevertheless, we consider it interesting that our tumor shared several common pathological features with OGNs; namely, a grossly elevated type, being located in the upper part of the stomach, and chief-cell dominancy in the OGN component [5, 13].

As described above, the intramucosal OGN component covered a limited area (< 5% of the whole tumor), and it was identified by embedding the whole advanced tumor into paraffin and careful microscopic examination. In contrast, the invasive component (also infiltrating the muscular and subserosal layers) was tubular adenocarcinoma which differed morphologically and immunohistochemically from OGN. We propose that the acquisition of the *TP53* mutation prompted such a phenotypic change in our tumor. Nevertheless, the morphology of the tumor cells in the invasive area and the focal MUC5AC expression therein were suggestive of a gastric phenotype. Therefore, we believe it is reasonable to assume that this advanced cancer originated from the OGN.

Given that we could not perform multi-region sequencing, ruling out the possibility of a collision tumor would be important. We believe the following two findings support the idea that our tumor is not a collision tumor. First, neighboring mucosal components with p53 wild-type and p53 aberrant patterns exhibited quite similar hematoxylin and eosin (H&E) morphology in terms of the size of glands. These components were difficult to distinguish from each other in loupe or low-power views. Second, the p53-aberrant mucosal component contained a minute area showing MUC5AC(-)/MUC6(+) beneath the predominant MUC5AC(+)/MUC6(-) area, just like the pattern of neighboring p53-wildtype mucosal OGN. It is not natural to assume that two independent mucosal tumors with MUC5AC-positive and MUC6-positive cells collided. However, due to the lack of multi-regional sequencing in this study, the possibility of an independent conventional adenocarcinoma adjacent to an OGN/GA-FGM-like lesion cannot be completely excluded.

### Conclusion

We presented a case in which an GA-FGM may have gained the potential for aggressive behavior through the acquisition of a *TP53* mutation. Our report sheds light on the possibility that the mutation of *TP53* contributes to OGN transformation into a more aggressive form of gastric cancer. This finding was enabled by careful histological assessment of the whole advanced cancer. Further cases are required to clarify the whole molecular mechanism of such histological transformations from OGN.

### Learning points

This case highlights several important lessons in the molecular pathological understanding of OGNs, entities which have recently been receiving increasing attention and recognition. First, this case offered novel insight into the OGN progression and broadened the current understanding of gastric tumor evolution. Through bulk DNA sequencing and double staining of p53/MUC5AC, it was suggested that *TP53* mutation acquisition is associated with aggressive transformation from OGN. Second, this case is in parallel with previous reports suggesting that GA-FGM, rather than GA-FG, may behave more aggressively. Finally, when diagnosing advanced gastric cancer, it is important to carefully assess the entire intramucosal tumor component microscopically. This allows for the discovery of underrecognized transformation pathways, even in gastric cancers that appear to show conventional histology.

## Supplementary Material

Suppl 1 Details of manual dissection of the tumor.

Suppl 2 List of genes contained in Axen Cancer Panel 1 for targeted DNA sequencing.

## Data Availability

The authors confirm that the data used in this work is available on reasonable request.

## Abbreviations

GA-FG: gastric adenocarcinoma of the fundic gland type
GA-FGM: gastric adenocarcinoma of the fundic-gland mucosa type
OGA: oxyntic gland adenoma
OGN: oxyntic gland neoplasm

## References

[1] Yao T, Vieth M, The WHO Classification of Tumors Editorial Board (2019). Oxyntic gland adenoma. WHO classification of tumors, digestive system tumors. 5th ed.

[2] Kushima R, Sekine S, Matsubara A, Taniguchi H, Ikegami M, Tsuda H (2013). Gastric adenocarcinoma of the fundic gland type shares common genetic and phenotypic features with pyloric gland adenoma. Pathol Int.

[3] Nomura R, Saito T, Mitomi H, Hidaka Y, Lee SY, Watanabe S, Yao T (2014). GNAS mutation as an alternative mechanism of activation of the Wnt/beta-catenin signaling pathway in gastric adenocarcinoma of the fundic gland type. Hum Pathol.

[4] Ushiku T, Kunita A, Kuroda R, Shinozaki-Ushiku A, Yamazawa S, Tsuji Y, Fujishiro M (2020). Oxyntic gland neoplasm of the stomach: expanding the spectrum and proposal of terminology. Mod Pathol.

[5] Ueyama H, Yao T, Akazawa Y, Hayashi T, Kurahara K, Oshiro Y, Yamada M (2021). Gastric epithelial neoplasm of fundic-gland mucosa lineage: proposal for a new classification in association with gastric adenocarcinoma of fundic-gland type. J Gastroenterol.

[6] Liu L, Zhang X, Fan X, Zhu X (2023). Genetic analysis of fundic gland-type gastric adenocarcinoma. Mol Clin Oncol.

[7] Miyazawa M, Matsuda M, Yano M, Hara Y, Arihara F, Horita Y, Matsuda K (2015). Gastric adenocarcinoma of fundic gland type: Five cases treated with endoscopic resection. World J Gastroenterol.

[8] Okumura Y, Takamatsu M, Ohashi M, Yamamoto Y, Yamamoto N, Kawachi H, Ida S (2018). Gastric adenocarcinoma of fundic gland type with aggressive transformation and lymph node metastasis: a case report. J Gastric Cancer.

[9] Ueyama H, Yao T, Nakashima Y, Hirakawa K, Oshiro Y, Hirahashi M, Iwashita A (2010). Gastric adenocarcinoma of fundic gland type (chief cell predominant type): proposal for a new entity of gastric adenocarcinoma. Am J Surg Pathol.

[10] Li C, Wu X, Yang S, Yang X, Yao J, Zheng H (2020). Gastric adenocarcinoma of the fundic gland type: clinicopathological features of eight patients treated with endoscopic submucosal dissection. Diagn Pathol.

[11] Zhang H, Wang S, Zhang Y, Ye F, Wang C (2022). Clinicopathological features of early stage gastric adenocarcinoma of fundic gland type: Case series. Medicine (Baltimore).

[12] Kimura K, Takemoto T (1969). An endoscopic recognition ofthe atrophic border and its significance in chronic gastritis. Endoscopy.

[13] Kim GH, Lee JS, Lee JH, Park YS (2024). Oxyntic gland neoplasms - from adenoma to advanced gastric cancer: a review of 29 cases. J Gastric Cancer.

[14] Wei K, Jiang L, Wei Y, Wang Y, Qian X, Dai Q, Guan Q (2015). The prognostic significance of p53 expression in gastric cancer: a meta-analysis. J Cancer Res Clin Oncol.

[15] Hidaka Y, Mitomi H, Saito T, Takahashi M, Lee SY, Matsumoto K, Yao T (2013). Alteration in the Wnt/beta-catenin signaling pathway in gastric neoplasias of fundic gland (chief cell predominant) type. Hum Pathol.

