# Supplementary material for: Transformation From Oxyntic Gland Neoplasm Into Advanced Gastric Cancer via TP53 Mutation: A First Case Report
Source: J Med Cases. 2026 Sep 4;17(10):546–53. doi: 10.14740/jmc5339 (PMC13568745; doi:10.14740/jmc5339)
Supplement: Supplementary file 1 [file jmc-17-10-546-s001.docx]

**Supplementary Material 1. Details of manual dissection of the tumor.**

The bulk tumor region within the dashed lines (i.e., the mucosal and invasive components) was dissected for DNA sequencing (scale bar: 5 mm). Two molecular pathologists certified by the Japanese Society for Pathology assessed the tumor cellularity, determining it to be 50%. Matched normal tissue was not analyzed.
