# Supplementary material for: Transformation From Oxyntic Gland Neoplasm Into Advanced Gastric Cancer via TP53 Mutation: A First Case Report
Source: J Med Cases. 2026 Sep 4;17(10):546–53. doi: 10.14740/jmc5339 (PMC13568745; doi:10.14740/jmc5339)
Supplement: Supplementary file 2 [file jmc-17-10-546-s002.docx]

**Supplementary Material 2.** List of genes contained in Axen Cancer Panel 1 for targeted DNA sequencing.

| *ABL1* | *ERBB3* | *HNF1A* | *NTRK1* |
| --- | --- | --- | --- |
| *AKT1* | *ERBB4* | *HRAS* | *NTRK2* |
| *AKT3* | *ERG* | *IDH1* | *NTRK3* |
| *ALK* | *ESR1* | *IDH2* | *PDGFRA* |
| *APC* | *ETV1* | *JAK1* | *PIK3CA* |
| *AR* | *ETV4* | *JAK2* | *PIK3R1* |
| *ATM* | *ETV5* | *JAK3* | *PPARG* |
| *AXL* | *EZH2* | *KDR* | *PTEN* |
| *BRAF* | *FANCA* | *KIT* | *PTPN11* |
| *BRCA1* | *FANCC* | *KRAS* | *RAF1* |
| *BRCA2* | *FANCF* | *MAP2K1* | *RB1* |
| *CCND1* | *FANCG* | *MAP2K2* | *RET* |
| *CDH1* | *FBXW7* | *MAP2K4* | *ROS1* |
| *CDK4* | *FGFR1* | *MET* | *RUNX1* |
| *CDK6* | *FGFR2* | *MLH1* | *SMAD4* |
| *CDKN2A* | *FGFR3* | *MPL* | *SMARCB1* |
| *CEBPA* | *FGFR4* | *MTOR* | *SMO* |
| *CSF1R* | *FLT3* | *MYC* | *SRC* |
| *CCNB1* | *FOXL2* | *MYCN* | *STK11* |
| *DDR2* | *GNA11* | *NOTCH1* | *TP53* |
| *EGFR* | *GNAQ* | *NPM1* | *VHL* |
| *ERBB2* | *GNAS* | *NRAS* | *WT1* |
